# Imaging mass spectrometry in drug development and toxicology

**DOI:** 10.1007/s00204-016-1905-6

**Published:** 2016-12-08

**Authors:** Oskar Karlsson, Jörg Hanrieder

**Affiliations:** 1 Center for Molecular Medicine, Department of Clinical Neuroscience, Karolinska Institute, 171 76 Stockholm, Sweden; 2 Department of Pharmaceutical Biosciences, Drug Safety and Toxicology, Uppsala University, 751 24 Uppsala, Sweden; 3 Department of Psychiatry and Neurochemistry, Sahlgrenska Academy at the University of Gothenburg, Mölndal Hospital, House V, 431 80 Mölndal, Sweden; 4 Department of Molecular Neuroscience, UCL Institute of Neurology, University College London, Queen Square, London, WC1N UK

**Keywords:** MALDI IMS, SIMS, BMAA, Toxins, Neurotoxicology, Preclinical, Biologics, Small-molecule drugs, 6-OHDA, L-DOPA

## Abstract

During the last decades, imaging mass spectrometry has gained significant relevance in biomedical research. Recent advances in imaging mass spectrometry have paved the way for in situ studies on drug development, metabolism and toxicology. In contrast to whole-body autoradiography that images the localization of radiolabeled compounds, imaging mass spectrometry provides the possibility to simultaneously determine the discrete tissue distribution of the parent compound and its metabolites. In addition, imaging mass spectrometry features high molecular specificity and allows comprehensive, multiplexed detection and localization of hundreds of proteins, peptides and lipids directly in tissues. Toxicologists traditionally screen for adverse findings by histopathological examination. However, studies of the molecular and cellular processes underpinning toxicological and pathologic findings induced by candidate drugs or toxins are important to reach a mechanistic understanding and an effective risk assessment strategy. One of IMS strengths is the ability to directly overlay the molecular information from the mass spectrometric analysis with the tissue section and allow correlative comparisons of molecular and histologic information. Imaging mass spectrometry could therefore be a powerful tool for omics profiling of pharmacological/toxicological effects of drug candidates and toxicants in discrete tissue regions. The aim of the present review is to provide an overview of imaging mass spectrometry, with particular focus on MALDI imaging mass spectrometry, and its use in drug development and toxicology in general.

## Introduction

Drug development requires detailed information of absorption, distribution, metabolism, excretion and toxicity (ADMET) properties of the novel drug candidates. Most drugs or metabolites are not uniformly distributed, and characterizing the tissue distribution of the drug candidate is key to understand other parts of drug development. Therapeutic agents need to be well distributed to their intended target site to have the desired pharmacological effect (Lanao and Fraile 2005; Mouton et al. 2008). Moreover, accumulation of parent drug or metabolites in unexpected tissues may lead to untargeted secondary pharmacology and toxicity (Castellino et al. 2011; Pellegatti and Pagliarusco 2011). Studies of compound distribution are not only important for preclinical safety assessment but also for mechanistic studies and risk assessment of environmental contaminants. Today most of our knowledge of drug or toxicant tissue localization is derived from whole-body autoradiography or homogenate LC–MS analysis (Castellino et al. 2011; McEwen et al. 2014). However, during the last decades imaging mass spectrometry (IMS) techniques that use various ionization modes such as desorption electrospray ionization (DESI), matrix-assisted laser desorption/ionization (MALDI), nanoparticle laser desorption/ionization (nano-PALDI) and secondary ion mass spectrometry (SIMS) have emerged as powerful alternatives for chemical imaging in situ (Hanrieder et al. 2015; Sugiura and Setou 2010; Waki et al. 2015).

**Fig. 1:**
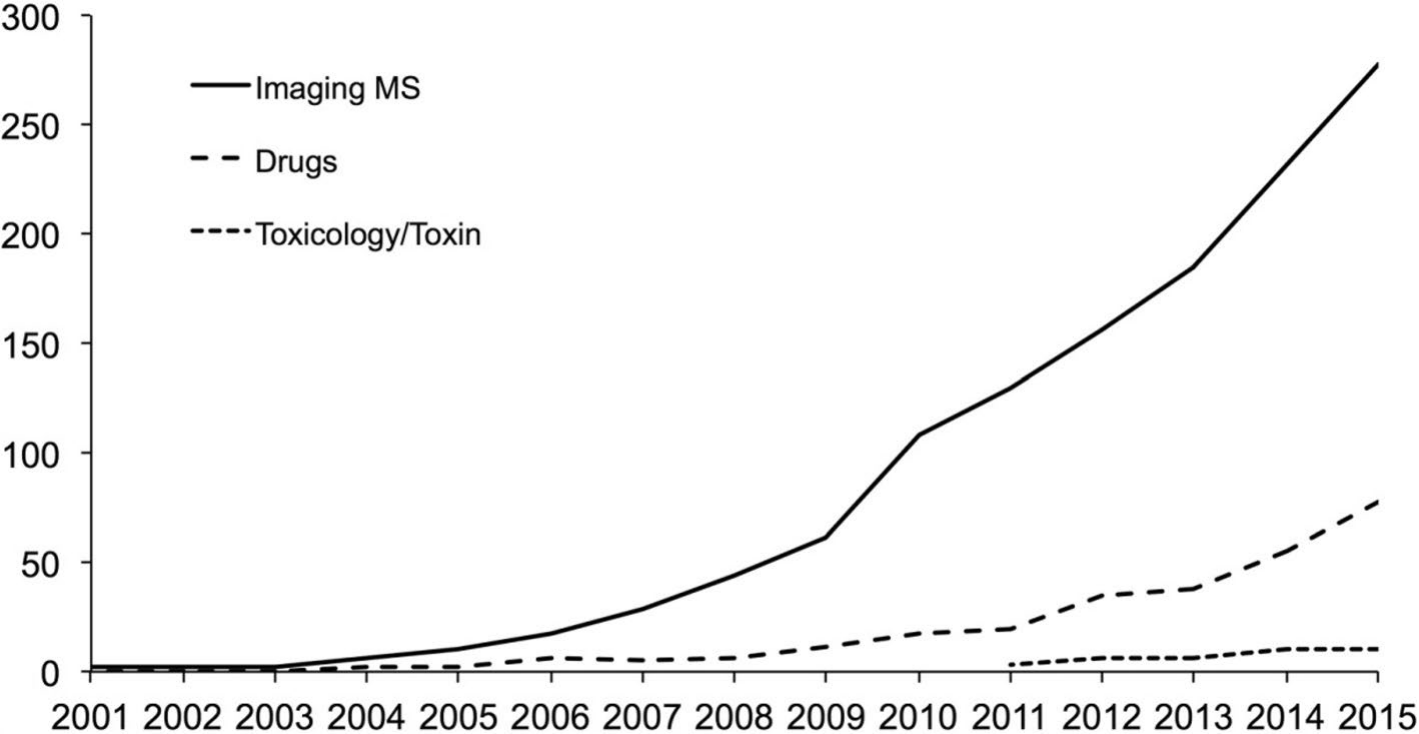
Publication statistics. Annual numbers of published articles concerning imaging mass spectrometry and its application to drug discovery and toxicology. Data retrieved from PubMed

Different imaging MS technologies have various strengths and limitations particularly with respect to spatial resolution and molecular information (Hanrieder et al. 2015). In contrast to whole-body autoradiography that images the localization of radiolabeled compounds, IMS provides the opportunity to simultaneously determine the discrete tissue distribution of the parent compound and its metabolites (Khatib-Shahidi et al. 2006; Solon et al. 2010; Stoeckli et al. 2007; Sugiura and Setou 2010). In addition, IMS features high molecular specificity and allows comprehensive, multiplexed detection and localization of hundreds of proteins, peptides and lipids in biological tissue samples (Cornett et al. 2007; McDonnell and Heeren 2007; Schwamborn and Caprioli 2010). IMS could therefore be used for omics profiling of pharmacological/toxicological effects of drug candidates and toxicants in discrete tissue regions. The increased relevance and popularity of IMS in biomedical and basic molecular biology research is reflected in the steady increase in IMS publications (Fig. 1). However, only few reports on using this technology for monitoring drugs, drug metabolites as well as drug safety and toxicological studies are available yet (Fig. 1), highlighting the challenges of employing IMS for such analyses due to e.g., matrix interference, isobaric interferences and sample throughput. The aim of the present review is to provide an overview of IMS, with particular focus on MALDI IMS, and its use in drug development and toxicology in general.

## Imaging mass spectrometry: principles and modalities

**Fig. 2:**
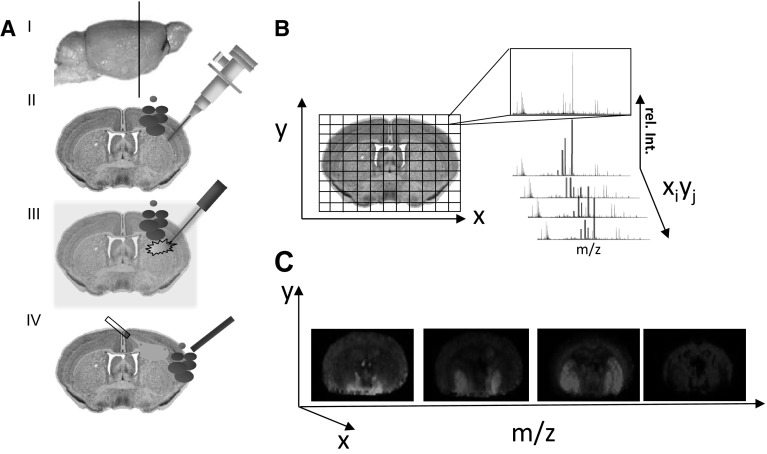
Principle of imaging mass spectrometry. **a** *I* Tissue sections are collected and mounted on a target for imaging mass spectrometry. *II* For SIMS IMS, tissue sections are probed with an ion beam, generating low molecular weight secondary ions (*m/z* > 1000 Da). *III* In contrast, MALDI IMS requires pre-coating with matrix (indicated in *yellow*) before systematic scanning with a laser probe. MALDI-based ionization generates larger intact molecular species, including peptides and proteins. *IV* In DESI IMS, a focused electro spray ionization probe is used to sample the tissue. The desorbed ions are collected with a co-aligned capillary that is connected to the mass spectrometer. **b** One mass spectrum is acquired for every *x*_i_, *y*_j_ coordinate of the scanned tissue section. **c** Single ion images are generated by mapping the intensity of an individual ion signal (*m/z*; rel.Int) over the whole tissue array

In IMS, the analyte localization is obtained by sequential acquisition of mass spectra from a predefined pixel array over a biological sample, i.e., tissue section or cell preparation (Fig. 2a, b). A major challenge in IMS is desorption and ionization of target species for subsequent MS analysis. Various imaging MS modalities have been developed over the last decades with MALDI, SIMS and DESI-based IMS being the most prominent. In SIMS, analytes are desorbed and ionized through impacting the sample with a focused ion beam, leading to formation and ejection of secondary ions (Colliver et al. 1997). In MALDI, a crystalline UV-absorbing matrix is used for enhancing analyte desorption and ionization through charge transfer in the gas phase (Hillenkamp et al. 1991; Karas and Hillenkamp 1988). In DESI, an electrospray is used for desorbing molecular species and generate ions, followed by transfer to the mass analyzer (Laskin et al. 2012; Takats et al. 2004). All ionization principles and IMS modalities have strengths and limitations, which have to be addressed by appropriate experimental design and by using the right technique for the right purpose.

In IMS, single ion images are generated by plotting the intensity pattern of a distinct molecular species over the analyzed tissue array (Fig. 2c). The spot-to-spot distance of the tissue array defines the pixel size and spatial resolution of the IMS analysis, respectively. The spatial resolution is mainly hampered by the probe—ion beam in SIMS, laser in MALDI and electrospray in DESI—but also lateral diffusion during sample preparation (MALDI) and analysis (DESI) has a major impact. In SIMS, spatial resolution below 1 μm and down to 30 nm is possible, but this is accompanied with compromises of the mass resolution and eventually the sensitivity (Benabdellah et al. 2010). Likewise, high spatial resolution MALDI IMS experiments of up to 1–10 μm are limited by the sensitivity of the method to the respective compounds of interest (Benabdellah et al. 2010). The spatial resolution may also be limited due to practical reasons including acquisition time bias and data size as there is a complete MS spectrum for each pixel in the acquired image. Analysis of complex imaging data can be performed using multivariate statistical analysis (MVSA) tools, such as principal component analysis (PCA) and maximum autocorrelation factor analysis (MAF). Multiple image analysis strategies have been reported for SIMS (Graham and Castner 2012; Henderson et al. 2009; Tyler et al. 2007) and MALDI IMS data (Deininger et al. 2008). This approach allows unbiased segmentation of a biological sample into distinct regions of interest by detecting variances and correlations in the multivariate data that are encompassed in the MVSA factors (PC or MAF). From the corresponding scores and loadings, the variables that are contributing the most (i.e., mass peak values) to these variances can be deduced revealing histology associated chemical changes.

## MALDI imaging mass spectrometry

The introduction of the soft ionization technique MALDI for mass spectrometry had a significant impact on the field of biological and biomedical sciences. This technology facilitates rapid and specific characterization of intact large biomolecular species, including proteins and peptides, which fueled the development of proteomics (Karas and Hillenkamp 1988). The technique is based on laser irradiation induced desorption and ionization of intact molecular species with the help of a crystalline UV-absorbing matrix (Fig. 2a). The generated ions are typically characterized with a time-of-flight (TOF) mass analyzer, but other mass analyzers such as Fourier transform ion cyclotron resonance (FTICR) or orbitrap can also be used. MALDI MS is characterized by its great mass range, sensitivity, high mass resolution and mass accuracy as well as its robustness, acquisition speed and insensitivity to sample impurities. MALDI IMS was introduced in 1997 by Caprioli et al. (1997). The technique is particularly well suited for medium to large biomolecules including glycolipids, neuropeptides and proteins. Spatial resolution in MALDI IMS is essentially dependent on three factors—the laser beam focus, the matrix crystal size and lateral diffusion of analyte molecules resulting during sample preparation. Efforts to push the spatial resolution with respect to the probe have been reported (Altelaar et al. 2007; Jurchen et al. 2005; Spengler and Hubert 2002). However, in large-scale experiments a practical spatial resolution of 10–20 μm may be achieved, but typically a 50–300 μm resolution is used. This can be compared to whole-body autoradiography, which has a spatial resolution around 5 μm when using film, and 50–100 μm when using phosphor detection methods for quantification (McEwen et al. 2014).

### Sample preparation

For MALDI IMS, the sample preparation significantly impacts the final data quality, particularly with respect to signal intensity, reproducibility, as well as spatial resolution. The sample preparation starts with tissue collection and storage, continues with tissue sectioning, sample wash and final matrix application. Each of these steps is critical for the final data quality. In particular, matrix application is a key aspect in the MALDI IMS experimental workflow and should be tailored according to numerous factors, including tissue origin and molecular target.

#### Tissue collection

For tissue imaging, adequate tissue collection is critical for data quality. Commonly used perfusion and fixation strategies are generally not compatible with mass spectrometry, due to interference of the polymeric fixation agents such as paraffin. Solutions to overcome this problem, including in situ trypsinization following paraffin removal or alternatively use of a reactive matrix 2,4-dinitrophenylhydrazine, have been suggested (Lemaire et al. 2007; Ly et al. 2016). Another elegant workaround was recently presented to delineate spatial protein N-glycosylation patterns (Gustafsson et al. 2015). These approaches, however, do not permit analysis of endogenous lipids, peptides and intact proteins due to washing steps with organic solutions (lipids, endogenous peptides) and enzymatic degradation (proteins). Fresh frozen tissues are therefore the most suitable and commonly used samples for IMS of all target species including low molecular weight compounds such as drugs, metabolites, and toxicants. A standardized protocol for quick and diligent tissue retrieval and snap freezing is essential, since postmortem delays by as little as 3-min result in severe degradation of proteins and peptides, truncating the correct representation of endogenous species and drug metabolites (Goodwin et al. 2008). Fresh frozen tissue samples are stored at −80 °C until preparation for IMS by cryosectioning. Tissue sections are collected with a cryostat microtome at −17 to −20 °C and thaw mounted onto conductive sample surfaces such as glass slides covered with indium tin oxide (ITO) or silicon wafers. The collected sections are dried immediately before storage at −80 °C in order to prevent damage by water condensation during freezing (Hanrieder et al. 2012).

#### Tissue washing

The choice of appropriate washing protocols is also a critical step in sample preparation. Lipid species typically do not require any advanced washing steps, whereas drugs, neuropeptides and proteins require optimized washing protocols for signal enhancement. These involve pH sensitive cleanup as well as organic solvents for precipitation of peptides and proteins while washing off remaining lipids that potentially interfere with the analyte signals prior to matrix application (Hanrieder et al. 2012; Shariatgorji et al. 2012a). Several washing protocols have been evaluated for enhancing protein signal in MALDI imaging. Stepwise washing with gradient alcohol has been found to give the most significant improvement in protein signal quality (Martin-Lorenzo et al. 2014; Seeley et al. 2008).

#### Matrix application

Different matrices are well suited for different compounds and the choice depends largely on the targeted analyte. A suitable MALDI matrix should be stable and enable ionization of the analyte without producing matrix-derived peaks in the m/z region of interest. Common matrices include α-cyano-4-hydroxycinnamic acid (CHCA), 2,5-dihydroxybenzoic acid (DHB), sinapinic acid (SA) and 9-aminoacridine (9-AA). The matrix should be applied in a standardized way that allows adequate extraction of the analyte while minimizing its delocalization. Several manual and automatic techniques have been used for matrix application including spotting, spray coating and sublimation, each with its own strengths and limitations with respect to simplicity, cost, resulting signal intensity, spatial resolution and signal reproducibility (Hanrieder et al. 2015).

### MALDI-based intact protein imaging

MALDI IMS is well suited for in situ protein analysis with retained spatial information. Although IMS is a powerful technique, there is still a need for complementary validation strategies to rule out false-positive findings as a result of experimental factors such as suppression effects and peak identification. Common validation approaches include immunohistochemistry, LC–MS/MS for proteomics and peptidomics in tissue extracts as well as in situ fragmentation (MS/MS) for top-down protein and peptide identification. A comprehensive list of identified neuropeptides and proteins observed in MALDI IMS experiments has previously been published (Hanrieder et al. 2015).

The application of MALDI IMS to projects of biological and clinical significance has increased since the introduction of the technique (Caprioli et al. 1997). However, many IMS publications are still of methodological character and are based on tissue sections from one or a few subjects. Some biological relevant experiments retrieve statistical information on different treatment groups directly from IMS data (Hanrieder et al. 2011, 2013; Jones et al. 2011; Karlsson et al. 2012, 2014; Kriegsmann et al. 2012; Ljungdahl et al. 2011; Meistermann et al. 2006; Onishi et al. 2013; Oppenheimer et al. 2010; Willems et al. 2010). Other studies rely on histology-directed profiling experiments to generate data for statistical analysis, followed by a single IMS experiment of one or two sections to elucidate the localization of interesting peptides and proteins (Burnum et al. 2009; Norris et al. 2007; Stauber et al. 2008; Uys et al. 2010; Yanagisawa et al. 2003). This may be a sensible rationale when the regions of interest are known prior to the experiment. By contrast, IMS can be used as an exploratory tool to localize affected organs or tissue regions (Hanrieder et al. 2013; Karlsson et al. 2012, 2014).

## MALDI imaging mass spectrometry for localization and quantification of drugs and toxicants

The major advantage of MALDI IMS for distribution studies is the possibility to simultaneously detect the parent molecule and its metabolites (Nilsson et al. 2015). However, detection of these small molecules is hampered by interference of matrix cluster ions. This limitation can be overcome by matrix-free laser desorption/ionization (LDI) approaches as well as other strategies including e.g., use of a stable isotope modified matrix, derivatization or MS/MS methodologies (Nilsson et al. 2010; Northen et al. 2007; Prentice et al. 2016; Shariatgorji et al. 2012b, 2015). The first experiment designed to study drug distribution using MALDI IMS in a whole-body mouse section was published in 2005 by Rohner et al. (2005). This approach was then extended for combined studies on drug/metabolite localization and mapping of endogenous biomarkers (Khatib-Shahidi et al. 2006). Quantification of target molecules by MALDI IMS is challenging but important for future integration of IMS in toxicological studies/evaluation. The sample preparation, in particular the matrix application, is essential for the data quality and reproducibility (see above). Moreover, when performing quantification of a target molecule in different organs or tissue regions ion suppression—mainly caused by different lipid and salt concentrations—could be a problem. To reduce the variability of IMS data, different strategies have been used to compensate for matrix crystallization effects and ion suppression during data acquisition. Approaches used include normalizing the spectra against the total ion count (TIC), other calculated normalization factors, or the signal intensity of an internal standard applied to the tissue section (Hamm et al. 2012; Hochart et al. 2014; Kallback et al. 2012; Stoeckli et al. 2007). The majority of published research concerns the quantification of small-molecule drugs (Chumbley et al. 2016; Groseclose and Castellino 2013; Koeniger et al. 2011; Nilsson et al. 2010). For example, the antibiotics rifampicin was quantified in pooled human plasma as well as in liver tissue from an animal dosed in vivo using a TOF/TOF instrumentation and a MS/MS approach with rifapentine as an internal standard. This enables multiple fragmentation events to be performed in a single laser shot, allowing the intensity of the analyte to be referenced to the intensity of the internal standard in each laser shot while maintaining the benefits of MS/MS (Prentice et al. 2016). Today, an increasing number of pharmaceutical companies are trying to develop therapeutic peptides or proteins including antibodies. A recent distribution study of the somatostatin analog octreotide shows that this synthetic octapeptide was clearly visualized and quantified by MALDI IMS (Takai et al. 2013). This suggests that IMS could be a suitable technique for ADMET studies not only for small-molecule drugs but also therapeutic peptides and other biological drugs.

The MALDI IMS methods developed for distribution and quantification of pharmaceutical drugs in tissues can also be applied for toxicants. For example, quantitative brain imaging of the environmental neurotoxin β-*N*-methylamino-l-alanine (BMAA)—suggested to be involved in neurodegenerative disorders (Cox et al. 2016; Spencer et al. 1987)—was performed after derivatization with 2,4-diphenyl-pyranylium (DPP) by relating the signals for BMAA in dosed rats a calibration curve constructed by spotting known concentrations of BMAA on a control tissue section (Shariatgorji et al. 2015). Moreover, it has been demonstrated that IMS can be used for quantification of the organochloride pesticide chlorodecone in the mouse liver (Lagarrigue et al. 2014). The method combines normalization by an internal standard in the matrix solution and the correlation with an orthogonal technique to achieve in situ absolute quantification (Lagarrigue et al. 2014). In addition, MALDI IMS has been used to study the brain distribution of the neurotoxic metabolite 1-methyl-4-phenylpyridinium (MPP+) after administration of 1-methyl-4-phenyl-1,2,3,6-tetrahydropyridine (MPTP) in mice (Kadar et al. 2014).

## Imaging mass spectrometry in mechanistic toxicology

Toxicologists traditionally screen for adverse findings by histopathological examination of tissues and monitoring selected biomarkers in urine or plasma. However, studies of the molecular and cellular processes underpinning toxicological and pathologic findings induced by drug candidates or toxicants are important to reach a mechanistic understanding and an effective risk assessment strategy (Ahuja and Sharma 2014). One of the strengths of IMS is the ability to directly overlay the molecular information from the mass spectrometric analysis with the tissue section and allow correlative visual comparisons of molecular and histologic information.

### Organ toxicology

Recent studies demonstrate the emerging role of IMS in the discovery of toxicity biomarkers and in obtaining mechanistic insights concerning toxicological mechanisms. For example, dabrafenib (DAB) is a drug approved for the treatment of specific tumors in adults. Preclinical studies—conducted to support the use of DAB in treatment of pediatric tumors—have shown drug-induced pathological effects on the kidney of juvenile rats. Tubular deposits were considered to be responsible for the renal pathogenesis due to effects of obstructive nephropathy (Groseclose et al. 2015). IMS analysis of juvenile rat kidneys contributed to a more thorough mechanistic understanding of the nephrotoxicity compared to LC–MS homogenate data alone, by characterizing tissue localization of DAB and its metabolites as well as determining the chemical composition of the renal deposits (Groseclose et al. 2015). The IMS results revealed that the tubular deposits consisted primarily of calcium phosphate and did not contain any drug-related material. This data allow a more complete risk assessment for pediatric treatment with DAB (Groseclose et al. 2015). MALDI IMS combined with data from manual tissue dissection analyzed by LC–MS/MS, and NMR has also been used to successfully identify the composition of renal deposits induced by two potential microsomal prostaglandin E synthase 1 (mPGES-1) inhibitors (Nilsson et al. 2012). The renal deposits consisted of their common metabolite bisulfonamide and an un-targeted analysis revealed molecular changes in the kidney that were specifically associated with the area of the tissue defined as pathologically damaged (Nilsson et al. 2012). Similarly, in a toxicological study of a drug candidate, MALDI IMS experiments revealed accumulation of the active drug in multiple organs where microcrystalline deposits were observed following administration of the pro-drug (Drexler et al. 2007). In a more recent study, MALDI IMS and DESI IMS were used to determine the composition of drug-induced crystal-like structures. PCA analysis of the MALDI IMS data revealed together with the MS/MS results that the crystal-like structures in the kidney of dosed rabbits are mainly composed of metabolites originating from demethylation and/or oxidation of the drug (Bruinen et al. 2016). In addition, the technique has been used to identify a transthyretin fragment (Ser28–Gln146) as a potential toxicity biomarker for the well-known nephrotoxicant gentamicin (Meistermann et al. 2006).

### Neurotoxicology

Many pathological processes are characterized not by large changes in the transcriptome or proteome, but by discrete changes in a subset of cells or specific loci. This may be particularly true for many pathophysiological processes in the brain (Hanrieder et al. 2015). The brain is highly organized in topographic maps and functionally related circuitries that might not be easily recognized or isolated for traditional proteomic analysis (Hanrieder et al. 2015; Karlsson et al. 2014). Hence, IMS may be particularly useful for mechanistic neurotoxicology. This is well illustrated by studies of the environmental neurotoxin BMAA and neurotoxin-based animal models of Parkinson’s disease (PD).

#### MALDI imaging mass spectrometry as a molecular exploratory tool: long-term effects of neonatal exposure to BMAA

**Fig. 3:**
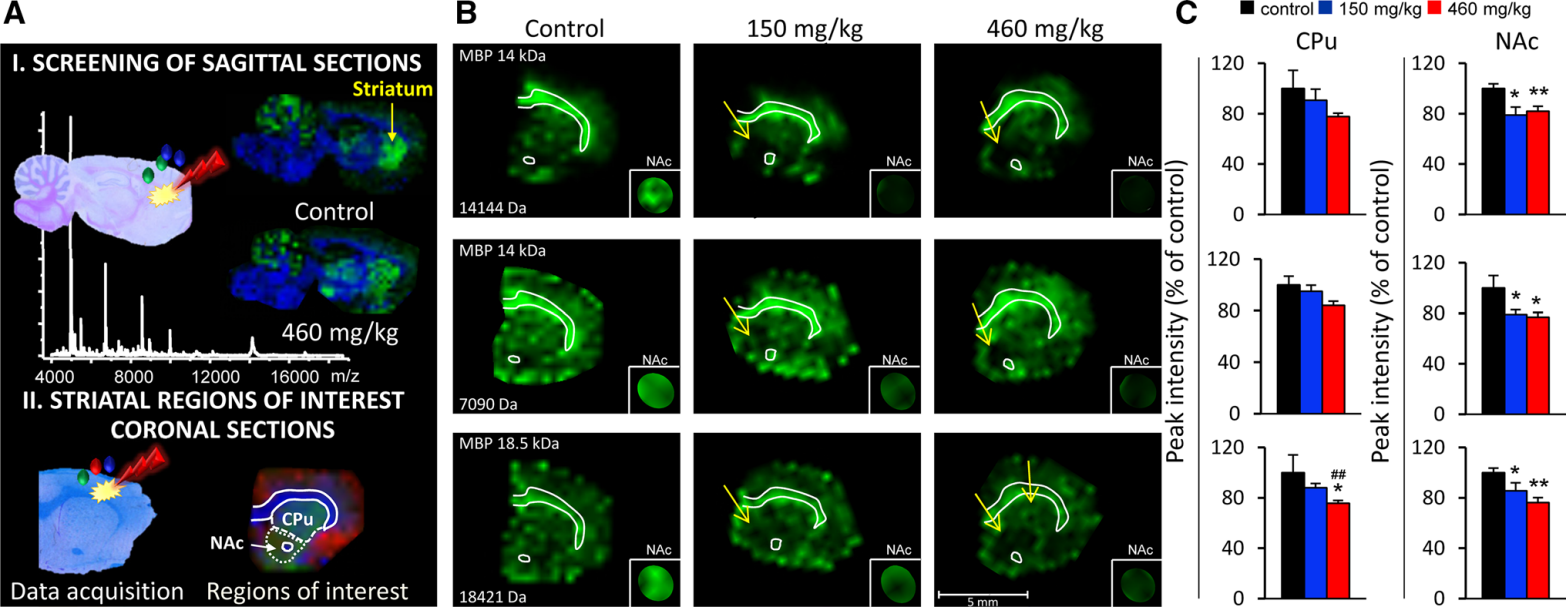
MALDI imaging mass spectrometry as a molecular exploratory tool in toxicology. Neonatal rats were treated with BMAA (150 and 460 mg/kg) or vehicle and their brains processed for MALDI IMS at adult age. **a** *I* To identify affected brain regions, an initial screening of sagittal brain sections from the left hemisphere of animals in the high treatment group and control group is performed. BMAA-induced changes in peak intensities are revealed in several brain areas. *II* Striatum is selected for further studies in a full-scale experiment and coronal striatal cryosections are obtained from the right hemisphere of all 21 animals. For statistical analysis, distinct regions of interest are determined. Histology as well as ion distribution images of several proteins are used to define striatum, divided into caudate putamen (CPu) and nucleus accumbens (NAc). **b** The analysis revealed a dose-dependent decrease in MBP levels in the CPu and NAc of adult rats neonatally treated with BMAA. The 14 kDa (both singly and doubly charged) and 18.5 kDa MBP isomers are shown in green. The arrows indicate the regional location of reduced protein expression. The small insertions are magnifications (×1.5) of NAc with the protein of interest displayed at higher color intensity and surrounding signals blocked by a mask. **c** The bar histograms show the peak intensity (% of control ± SEM) for the selected proteins in CPu and NAc. *p < 0.05, **p < 0.01 compared with vehicle control animals, ^##^ *p* < 0.01 compared with 150 mg/kg (Kruskal–Wallis test and Mann–Whitney *U* test). Control *n* = 6; BMAA 150 mg/kg *n* = 8; 460 mg/kg *n* = 7. Adapted from: Karlsson et al. Mol Cell Proteomics. 2014; 13(1):93–104

BMAA is transferred via breast milk to nursed offspring (Andersson et al. 2013, 2016) and demonstrated to induce long-term cognitive impairments after neonatal exposures of rats (Karlsson et al. 2009a, b, 2011). MALDI IMS has been used as an exploratory tool in combination with histopathology to identify brain regions affected by the neonatal BMAA administration (Fig. 3a). An initial screening of sagittal sections from the left hemispheres of rat brains collected 6 months after toxin exposure revealed BMAA-induced protein changes in several brain regions including the striatum and hippocampus (Karlsson et al. 2014). These regions were selected for full-scale experiments using coronal cryosections from the right hemispheres of all 21 animals (Karlsson et al. 2012, 2014). The striatum was divided into caudate putamen (CPu) and nucleus accumbens (NAc) and the regional analysis revealed 10 and 18 significantly altered protein peaks in the CPu and the NAc of both high- and low-dose groups compared to controls. Decreased levels of myelin basic protein (MBP) were demonstrated (Fig. 3b, c) and indicate that developmental exposure to BMAA could induce structural effects on axonal growth and/or directly on the proliferation of oligodendrocytes and myelination, which might be important for the observed long-term cognitive impairments (Karlsson et al. 2014). The IMS analysis of hippocampus revealed a decreased expression of proteins involved in energy metabolism and intracellular signaling in the adult hippocampus of both dose groups (Karlsson et al. 2012). In addition, the high BMAA dose induced changes in the expression of S100β, histones, calcium- and calmodulin-binding proteins and guanine nucleotide-binding proteins. Interestingly, at this dose also severe lesions in the adult hippocampal CA1 region including neuronal degeneration, cell loss, calcium deposits and astrogliosis were observed (Karlsson et al. 2012). Ultrastructural examination by transmission electron microscopy revealed intracellular deposition of abundant bundles of closely packed parallel fibrils in neurons, axons and astrocytes of the CA1. SIMS IMS and laser capture microdissection followed by LC–MS/MS showed enrichment of specific phospholipids and proteins in this brain region (Hanrieder et al. 2014a, b; Karlsson et al. 2015a, b). These molecular studies of BMAA-induced effects further illustrate the usefulness of combining IMS with other experimental techniques to identify target regions and investigate toxicological events.

#### Imaging of neurotoxin-based animal models mimicking Parkinson’s disease and L-DOPA-induced dyskinesia

Understanding the molecular pathways that underlie neurodegenerative diseases is an ongoing challenge in which MALDI IMS can prove to be helpful. Several animal models of neurodegenerative disease, particularly PD, are based on injection of neurotoxins such as MPTP or 6-OHDA for selective degeneration of nerve cells mimicking neurodegeneration and impairment of distinct neuronal circuits (Karlsson and Lindquist 2016; Ungerstedt 1968). MALDI IMS has been used to validate the reduction in the calmodulin-binding protein PEP-19 and determine its brain distribution in the MPTP mouse model of PD (Skold et al. 2006). In addition, in situ mass spectrometry based profiling has been used to delineate spatial molecular changes in the striato-nigral circuit following 6-OHDA injection of mice (Pierson et al. 2004). Similarly, endogenous peptide levels were outlined in rat brain using MALDI IMS in a 6-OHDA rat model of PD (Hanrieder et al. 2011, 2012; Ljungdahl et al. 2011, 2013). Detection and quantification of endogenous neuropeptides in situ is challenging as commonly used antibody-based techniques are hampered by several factors including throughput, quantification and specificity. Antibody-based techniques require a priori knowledge of the target species and are limited in terms of throughput as it only allows detection of few species at the same time. Most importantly, immunohistochemistry of neuropeptides is significantly hampered by antibody specificity, which is particularly relevant for opioid peptides. These peptide species differ in only a few C-terminal amino acids, which compromise the reliability of immunohistochemistry results significantly. In addition, immunohistochemistry provides only semi-quantitative information. In contrast, neuropeptidomics approaches using LC–MS on tissue extracts—despite being a powerful approach for endogenous peptide characterization (Karlsson et al. 2013; Nilsson et al. 2009)—are limited in that the dissection and tissue extraction result in loss of spatial information. This highlights the need for a molecular imaging technique to comprehensively delineate neuropeptide regulations in situ something that can be achieved by using IMS.

**Fig. 4:**
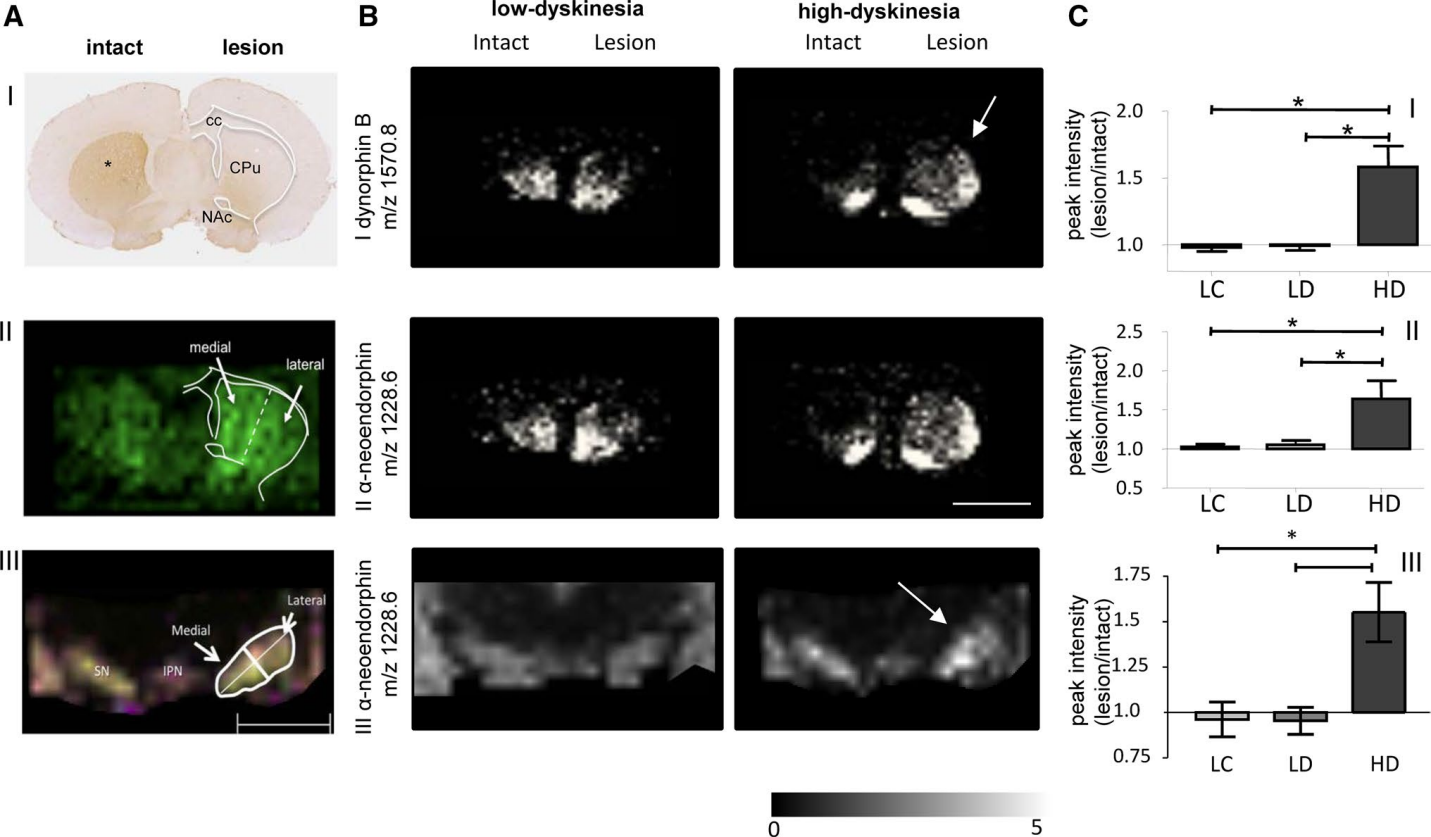
MALDI imaging mass spectrometry analysis of neuropeptides in L-DOPA-induced dyskinesia. **a** *I* Unilateral 6-OHDA injection leads to dopamine depletion (illustrated by tyrosine hydroxylase immunostaining*). L-DOPA therapy results in two distinct groups with low and high dyskinesia. *II* Striatal and *III* substantia nigra sections are analyzed by MALDI IMS and regions of interest (ROI) are assigned for spectral data extraction. **b** Dynorphin peptides, *I* dynorphin B and *II* alpha neoendorphin were significantly increased in the dorsolateral striatum of high (HD) compared to low dyskinetic (LD) animals and lesion controls (LC). *III* Similarly, endogenous dynorphin peptides (e.g., alpha neoendorphin) were increased in the substantia nigra reticulata on the parkinsonian side following L-DOPA treatment. **c** The results are verified by spectral analysis and nonparametric ANOVA and post hoc statistics. *Scalebar* 2 mm. Adapted from: Ljungdahl et al. PLoS One. 2011; 6(9) and Hanrieder et al. Mol Cell Proteomics. 2011; 10(10)

L-DOPA-induced dyskinesia (LID) is a form of dyskinesia that occurs in up to 80% of all PD patients after 5–10 years of L-DOPA treatment (Ahlskog and Muenter 2001). MALDI IMS has been employed to characterize spatial regulations of dynorphin opioid peptides in LID using the PD model based on unilateral 6-OHDA lesions in rat brain (Fig. 4) (Hanrieder et al. 2011; Ljungdahl et al. 2011). In this model, animals develop PD pathology only on one side and the other side can serve as an internal control (Ungerstedt 1968). This offers an elegant solution to avoid the effects of intra-sample group variation, which is particularly relevant IMS and mass spectrometry in general as this approach accounts for variation induced by suppression effects. Following 6-OHDA lesion and L-DOPA treatment, MALDI imaging revealed that dynorphin B and alpha neoendorphin were significantly elevated in the dorsal lateral striatum in the high dyskinetic group but not for low dyskinetic animals (Fig. 4b I–II) (Hanrieder et al. 2011). In addition, both dynorphin species correlated positively with LID severity (Fig. 4c I–II). Similarly, the dynorphin peptides were elevated in the substantia nigra that constitutes the main output structure of the striatal projections in the direct pathway of motor control (Fig. 4a–c III) (Ljungdahl et al. 2011).

## Concluding remarks

In drug development, information about the in vivo distribution of a drug candidate after its administration is essential as it facilitates the understanding of the mechanisms underlying the efficacy or toxicity of the drug. Despite recent advances in IMS methodologies including MALDI IMS, whole-body autoradiography based on the method of Ullberg published more than 60 years ago (Ullberg 1954, 1977) still remains the gold standard for determining tissue distribution of drugs (McEwen and Henson 2015; McEwen et al. 2014). One of the strengths of IMS, compared to whole-body autoradiography, is the opportunity to simultaneously determine the discrete tissue distribution of the parent compound its metabolites as well as endogenous molecules. This raises the exiting possibility of performing experiments that monitor both distribution and pharmacological/toxicological mechanisms at the same time. However, IMS of small molecules is limited by endogenous and matrix-related overlapping (isobaric) peaks. The instrument must therefore be able to differentiate the target compounds from the background noise. This is usually achieved by performing the experiments in MS/MS mode, which impairs a simultaneously and untargeted analysis of multiple analytes. Future developments in instrument performance and methodologies are needed to simplify the experimental procedure, provide reliable quantitative data and to streamline on tissue omics profiling. At present, IMS could be considered as a complementary method that may give valuable information during preclinical studies of drug candidates as well as in toxicological research. As the work with technical improvements is continuously ongoing, IMS could be expected to become a standard method in drug development and toxicology (Fisher et al. 2016; Ogrinc Potocnik et al. 2015; Prentice et al. 2016).
